# Atypical functional connectivity in resting-state networks of individuals with 22q11.2 deletion syndrome: associations with neurocognitive and psychiatric functioning

**DOI:** 10.1186/s11689-016-9135-z

**Published:** 2016-01-21

**Authors:** Leah M. Mattiaccio, Ioana L. Coman, Matthew J. Schreiner, Kevin M. Antshel, Wanda P. Fremont, Carrie E. Bearden, Wendy R. Kates

**Affiliations:** Department of Psychiatry and Behavioral Sciences, State University of New York Upstate Medical University, Syracuse, 13210 NY USA; Department of Psychiatry and Biobehavioral Sciences and Neuroscience Interdepartmental Program, University of California Los Angeles, Los Angeles, 90095 CA USA; Department of Psychology, Syracuse University, Syracuse, 13244 NY USA

**Keywords:** 22q11.2 deletion syndrome, Resting-state fMRI, ICA, Schizophrenia

## Abstract

**Background:**

22q11.2 deletion syndrome (22q11DS) is a neurogenetic condition associated with deficits in neuropsychological functioning and psychiatric disorders. This deletion confers a high risk for the development of psychosis, as approximately 30–45 % of individuals develop psychosis in adulthood. Previous reports of resting-state functional magnetic resonance imaging (rs-fMRI) functional connectivity patterns in 22q11DS have demonstrated that atypical connectivity is associated with both the emergence and severity of psychotic symptoms. However, due to sample overlap and large age ranges of samples spanning multiple critical periods of brain maturation, more independent studies with samples within the window of time when psychotic symptoms have been shown to emerge (ages 17–26) are needed. Resting-state networks (RSNs) in 22q11DS during this stage of brain development may thus provide insight into the dynamic changes in functional integration that influence the incidence of prodromal symptoms and neurocognitive deficits characteristic of this syndrome.

**Methods:**

Independent component analysis (ICA) was performed to identify RSNs in a combined sample of 55 individuals with 22q11DS (27 males; age range 17–26) and 29 controls (17 males; age range 17–23, consisting of 8 siblings without the deletion and 21 typically developed individuals) from two research sites. We conducted a full factorial analysis to determine group differences between 22q11DS and controls. A Poisson regression analysis was conducted in the 22q11DS group to determine relationships of rs-fMRI network connectivity with psychiatric symptoms based on factors of the 18-item Brief Psychiatric Rating Scale. Nonparametric Spearman correlations were performed to test associations between within-network functional connectivity (FC) and performance on measures of verbal memory (California Verbal Learning Test) and executive function (Behavior Rating Inventory of Executive Function Adult version) in 22q11DS.

**Results:**

Between-group network connectivity analyses revealed significant differences in 9 RSNs. Decreased network FC in 22q11DS was observed in the following networks: high-level visual processing network (HLVPN), low-level visual processing network (LLVPN), visual/precuneus network, left frontal-parietal network (LFPN), right frontal-parietal network (RFPN), and self-referential network (SRN). In contrast, greater network FC in 22q11DS was observed in subclusters of the LLVPN, visual/precuneus network, limbic network (LN), default mode network (DMN), and visuospatial processing network (VSPN). Increased functional connectivity of the right cuneus (visual/precuneus network) and right superior parietal lobule (DMN) in 22q11DS was positively associated with both thought disturbance and disorganization factors of the Brief Psychiatric Rating Scale (BPRS). Decreased functional connectivity in the left posterior cingulate (LLVPN) was associated with higher thought disturbance scores in 22q11DS. No associations with our neurocognitive measures passed correction for multiple comparisons (Bonferroni-corrected *p* ≤ 0.0014).

**Conclusions:**

Our findings suggest that atypical network connectivity within RSNs may be indicative of increased risk for developing psychosis and supports the utility of RSNs as biomarkers of prodromal symptoms in 22q11DS.

**Electronic supplementary material:**

The online version of this article (doi:10.1186/s11689-016-9135-z) contains supplementary material, which is available to authorized users.

## Background

Over the last two decades, resting-state functional magnetic resonance imaging (rs-fMRI) studies of individuals with schizophrenia have revealed correlations between aberrant functional connectivity and psychotic symptoms and/or cognitive deficits [1, 2]. These findings may inform the mechanisms underlying the emergence of psychotic symptoms in 30–40 % of individuals with 22q11.2 deletion syndrome (22q11DS) [3, 4], a genetic syndrome that occurs in 1:2000–1:6000 live births [5, 6], and confers the highest known risk for schizophrenia apart from having a monozygotic twin with the disorder [7–9]. Childhood psychiatric disorders are highly prevalent [9, 10, 11], followed by the emergence of prodromal or overt psychosis in late adolescence and adulthood [12, 13–16]. The neurocognitive phenotype includes deficits in verbal learning and memory, visuospatial cognition, working memory, and executive functioning [16, 17].

Psychosis has been characterized as the abnormal functional integration of brain processes [18]. In fMRI, functional integration is apparent when spatially independent brain regions are co-activated in either the presence of a goal-directed task or while the brain is at rest [19, 20]. In the past, many functional imaging studies of individuals with 22q11DS have utilized task-based paradigms, which can be disadvantageous in clinical populations of individuals who cannot complete the task [21]. In contrast, rs-fMRI characterizes functional connectivity (FC) of brain regions at rest. Both independent component analysis (ICA) and seed-based approaches have provided a canonical set of resting-state networks (RSNs) related to visual and sensorimotor processing, executive functioning, and a default mode network (DMN); the latter is involved in internal mentation and memory that increases in activity when the brain is not engaged in overt cognitive tasks [20, 22–26].

The majority of studies in schizophrenia [1, 2, 19, 21, 27] point to decreased FC within the DMN. Moreover, increased connectivity within RSNs subserving emotional and sensory processes has been linked to the presence of visual and auditory hallucinations, [28–30]. Thus, converging evidence from studies of idiopathic schizophrenia supports the notion that aberrant connectivity patterns found in RSNs may play a role in understanding the occurrence of psychotic symptoms in individuals with 22q11DS.

The first study to describe RSNs in 22q11DS, based on a sample aged 12–19, reported both increased and decreased FC in the DMN, sensorimotor, visuospatial, and higher-level visual networks [31] in patients with 22q11DS relative to controls. Moreover, in 22q11DS, an association was observed between DMN activity and prodromal symptoms. A subsequent study from the same group, which used a support vector machine classifier and was based on a sample aged 12–22 that overlapped with the original report, found differences in frontal, cingulate, and temporal connectivity between individuals with 22q11DS and typically developing controls [32]. These findings were also significantly correlated with IQ in the 22q11DS group [32]. A third study from the same group investigated associations between resting-state functional and structural connectivity with age and psychosis [33]. They observed reductions in both structural and functional connectivity between the anterior and posterior nodes of the DMN, as well as the anterior region of the DMN and left inferior parietal lobe (IPL). No significant associations with prodromal symptoms were found [33]. However, this group’s combined sample contained a large age range (8–28 in 22q11DS and 6–25 in controls) that was split into three relatively small age groups. Finally, several investigators who collaborated on the present report used a seed-based approach to study FC of the posterior cingulate within the DMN and social reciprocity; reporting that increased FC between the posterior cingulate, anterior cingulate, and medial prefrontal cortex was associated with higher social competency [34].

These findings suggest that FC is aberrant in 22q11DS and may underlie both psychiatric and cognitive impairments in individuals with this syndrome. However, three of the aforementioned studies were from the same group of investigators, featured considerable overlap in sample participants, and were based largely on the same scans [31–33]. Thus, more studies are needed involving independent samples. Previous studies also used samples with large age ranges, potentially confounding results as brain development is markedly different from childhood, to adolescence, to adulthood [35]. Accordingly, the investigation of RSNs as biomarkers of psychosis warrants further studies involving larger, multi-site samples within a smaller age group. Elucidating functional connectivity patterns characteristic of brains aged between 17 and 26, a time critical in the development of prodromal symptoms and conversion to psychosis [36], may serve to highlight alterations in RSNs and the ways in which functional interactions may influence the transition into the prodromal state.

Here, we combined data from two research sites to investigate rs-fMRI data in 55 adolescents and young adults with 22q11DS and 29 typically developing/sibling controls by utilizing independent component analysis (ICA), a data-driven method to identify spatially independent components that are temporally correlated [23]. Our goals were (1) to determine differential functional connectivity patterns between 22q11DS and control groups and (2) to determine whether these within-network connectivity patterns are associated with symptoms of psychosis and neuropsychological functioning in 22q11DS. We hypothesized that atypical FC patterns in the DMN, specifically regions in the medial prefrontal and temporal lobe, would be associated with the presence of prodromal symptoms. We also hypothesized that resting-state networks involving frontal-parietal regions would be associated with neuropsychological functioning.

## Methods

### Participants

Data were acquired from large-scale longitudinal studies of risk factors for psychosis in 22q11DS that are being conducted at two academic medical centers, SUNY Upstate Medical University, Syracuse, NY, and University of California, Los Angeles, CA (UCLA). Data consisted of 55 participants with 22q11DS (SUNY: 39; 19 males; mean age 20.47, SD 2.05; 29 right handed; UCLA: 16; 8 males; mean age 20.31, SD 2.94; 15 right handed), and 29 controls comprised of both siblings without the deletion and typically developed community controls (SUNY: 25; 15 males; mean age 20.70, SD 1.22; 25 right handed; UCLA: 4; 2 males; mean age 19.0, SD 1.83; 4 right handed). Independent *t* tests between siblings and controls were conducted for age, gender, and full scale IQ, where *p > 0.05.* Therefore, since siblings did not differ in demographic measures, we combined them into one control group. All participants from both subsamples were native English speakers.

Exclusion criteria for the SUNY sample included seizure disorder, fetal exposure to alcohol or drugs, parent-reported elevated lead levels or birth weight under 2500 grams, loss of consciousness lasting longer than 15 minutes, paramagnetic implants, or orthodontic braces. Potential controls with a personal or family history of schizophrenia or bipolar disorder were also excluded. Exclusion criteria for the UCLA subsample included additional neurological or medical condition that could affect imaging measures, insufficient fluency in English, substance or alcohol abuse and/or dependence within the last 6 months, and any condition that is a contraindication for MRI acquisition. Additional details of exclusion criteria for the UCLA subsample can be found in [37]. Details of this sample have been described elsewhere [34]. Additionally, our control group did not meet criteria for a psychotic disorder, based on information gathered from the administration of the Structured Clinical Interview for DSM-IV Axis I Disorders (SCID; [38]) for both sites. Diagnosis of 22q11DS in the SUNY subgroup was confirmed by fluorescence in situ hybridization (FISH). In the UCLA subgroup, 22q11DS diagnosis was confirmed by either FISH or array comparative genomic hybridization (CGH).

Within the combined 22q11DS group, 24 were treated with one or more antipsychotic, stimulant, antidepressant, antianxiety, or mood stabilizing medications at the time of their scan. Two controls were treated with an antidepressant, antianxiety, or stimulant medication at the time of their scan. Due to the fact that data were taken from an ongoing longitudinal study, control participants in the SUNY study who presented with an anxiety disorder were excluded from the first timepoint; however, those who later developed an anxiety disorder were not. (The current report is based on data acquired at the fourth timepoint of the SUNY study.) Controls with ADHD/learning disability were not excluded from the SUNY sample at any timepoint, in order to maximize comparability between the control sample and the higher functioning participants in the 22q11DS group. Based on *t* tests, demographic variables did not differ significantly between sites for either patients or controls. Table 1 contains information regarding demographics and medication for all 84 participants included in our group analysis. Additional information regarding participant characteristics between sites can be found in Additional file 1: Table S1. The institutional review boards of SUNY Upstate Medical University and UCLA approved all study procedures, and all participants from both sites provided informed consent or assent.

**Table 1:** Demographic and medical data

	22q11DS	Controls	*P* value
	*N* = 55	*N* = 29	
Age^a^	20.42 (2.31)	20.46 (1.41)	0.923
Gender (male, %)	27 (49.1 %)	17 (58.6 %)	0.412
Full scale IQ^a^	73.35 (10.51)	109.45 (9.72)	<0.001
Psychiatric diagnosis, *n* (%)			
Psychotic disorder	5 (9.10 %)	0 (0 %)	0.024
ADHD	9 (16.36 %)	3 (10.34 %)	0.460
Anxiety disorder	15 (27.27 %)	3 (10.34 %)	0.046
Mood disorder	9 (16.36 %)	1 (3.45 %)	0.037
Current medication*, n* (%)			
Antipsychotic/mood stabilizer	7 (12.73 %)	0 (0 %)	0.007
Antidepressant/antianxiety	18 (32.73 %)	1 (3.45 %)	<0.001
Stimulants	6 (10.91 %)	2 (6.90 %)	0.557

Demographic and medical data for participants in our group analyses; from our initial sample of 87, 2 patients were excluded due to image quality or age and 1 control was excluded due to lack of neuropsychological data

^a^Mean and standard deviation are provided for age and full scale IQ. Independent *t* tests were conducted to determine differences between 22q11DS and control samples. Demographics did not differ across sites. Additional information can be found in Additional file 1: Table S1

### Neuropsychological and psychiatric assessment

Participants underwent neuropsychological testing by a trained, master’s- or doctoral-level psychologist at both sites (see [39, 40] for more details about training and quality assurance for the UCLA site). General intellectual functioning was assessed with the Wechsler Adult Intelligence Scale, Third Edition, [41] in the SUNY subsample and the Wechsler Abbreviated Scale of Intelligence (WASI; [42]) in the UCLA subsample. The California Verbal Learning Test Adult Version, (CVLT-A; [43]) was used in both cohorts to assess verbal memory. Since versions differed between sites, raw scores from list A were extracted for the present analyses. To assess executive function, the Behavioral Rating Inventory of Executive Function-Adult Version Informant Report Form (BRIEF-A; [44]) was given to the parents of the participants from both sites to complete. For our correlation analysis, we used the Global Executive Composite score (GEC), which is a composite score from the Behavioral Regulation and Metacognition Indices. We performed between-site *t* tests on raw scores from the CVLT and GEC scores, revealing no significant differences between sites for either measure. Both the CVLT and BRIEF-A had normal distributions for both groups of the combined sample, and *p* values are provided in Additional file 2: Table S2.

A trained master’s- or doctoral-level psychologist or psychiatrist administered psychiatric assessments at each site. To determine the presence of psychiatric conditions present in the aforementioned sample, both sites utilized the Structured Clinical Interview for DSM-IV Axis I Disorders (SCID; [38]). The 18-item Brief Psychiatric Rating Scale (BPRS; [45]) was used to examine symptoms associated with schizophrenia. Individual items from the BPRS were summed based on previous studies of factor analyses of the symptoms assessed in the BPRS [46, 47]. Specifically, we conducted Poisson regression analyses on four factors: thought disturbance (which consisted of the items grandiosity, suspiciousness, hallucinatory behavior, and unusual thought content); negative symptoms (consisting of emotional withdrawal, motor retardation, uncooperativeness, and blunted affect); affect (consisting of somatic concern, anxiety, guilt feelings, depressive mood, and hostility); and disorganization (consisting of the items conceptual disorganization, tension, mannerisms and posturing, and disorientation) (Table 2).

**Table 2:** Means (SD) of psychiatric and neuropsychiatric measures

	22q11DS	Controls	*P* value
BPRS^a^			
Thought disturbance	6.11 (4.30)	4.07 (0.37)	0.001
BPRS			
Disorganization	5.49 (3.39)	4.14 (0.44)	0.005
BPRS			
Affect	8.60 (3.73)	6.86 (2.08)	0.008
BPRS			
Negative symptoms	6.69 (3.27)	4.86 (1.36)	0.001
CVLT^ab^			
Raw score	38.62 (11.30)	54.31 (7.71)	<0.001
BRIEF-A informant report^a^			
Global executive function index	63.15 (12.16)	44.88 (7.78)	<0.001

^a^Brief Psychiatric Rating Scale 18-item; California Verbal Learning Test Adult version; Behavioral Rating Inventory of Executive Function for Adults

^b^The CVLT raw score includes combined scores from list A. Independent *t* tests were computed between 22q11DS and controls

### FMRI data acquisition

Images from the SUNY subsample were acquired with a 3 Tesla Siemens Tim Trio syngo MR B17 with an 8-channel head coil receiver (Siemens Medical Solutions, Erlangen, Germany). A resting-state functional scan lasting approximately 5 min included 152 Blood Oxygen Level Dependent (BOLD) images (34 axial slices, 4 mm thickness, no gap) using an ep2d_bold sequence (with the following parameters: repetition time [TR] = 2000 ms, echo time [TE] = 30 ms, voxel size = 4.0 × 4.0 × 4.0, flip angle = 90°, acquisition matrix = 64 × 64, field of view [FOV] = 256). Images from the UCLA subsample were acquired with a 3 Tesla Siemens Tim Trio scanner, using a 12-channel head coil receiver. The resting-state scan lasted 5 min and consisted of 152 BOLD 3D images: voxel size 3.0 × 3.0 × 4.0 mm, echo time [TE] = 30 ms, repetition time [TR] = 2000 ms, echo spacing = 0.79 ms, 34 axial slices, slice thickness 4.0 mm, slice spacing 0 mm, flip angle 90°, field of view [FOV] = 192, matrix size = 64 × 64. Participants from both sites were instructed to keep their eyes open and avoid falling asleep.

### Preprocessing of fMRI data

Data for the combined sample were preprocessed using Statistical Parametric Mapping (SPM5; Wellcome Trust Centre for Neuroimaging, 2005, London, UK, http://www.fil.ion.ucl.ac.uk/spm/). BOLD images were first visually inspected to ensure the absence of major artifacts (i.e., significant signal dropout, excessive noise, ghosting). This procedure was then repeated throughout various stages of the preprocessing. Images were then motion corrected using INRIalign [48], which is an algorithm unbiased by local signal changes. Participants were excluded if raw BOLD images contained any of the artifacts mentioned above or if they had too much motion based on the following criteria: more than 2 mm across the entire run and rotation greater than 2°. One patient from the SUNY subsample was excluded due to a significant signal dropout in the raw BOLD images; no other participants in the combined sample were excluded due to either motion or image artifacts. Motion adjustment, an alternative to adding motion regressors to the design matrix, was then carried out using ArtRepair. This algorithm suppresses the residual fluctuations due to interpolation errors from large motions [49]. Subsequently, a despiking function was applied to remove spikes caused by motion. By applying these steps in the processing pipeline, variation is removed from the realigned time series [49]. Spatial normalization was then performed on the motion-corrected images into Montreal Neurological Institute Space. Voxels were resampled at 3 mm^3^ using trilinear interpolation. Preprocessing was completed with Gaussian spatial smoothing with a kernel of 6 mm full width half maximum (FWHM).

### Component and network identification

Group-level spatial independent components analysis was performed using the Infomax algorithm in the GIFT toolbox (available at http://mialab.mrn.org/software/gift/index.html). Our group-level analysis consisted of both patients and controls processed together. Subject-specific principal component analysis was run based on the expectation maximization algorithm. Using the Infomax algorithm, group-level analysis was run 20 times in ICASSO. Instead of manually restricting the components, we allowed the algorithm to estimate the optimal number of components for our data. Each component represents non-overlapping spatial patterns corresponding to a specific time course derived from the group data set [50]. The algorithm estimated 33 components, which were then visually inspected to determine their classification as a network or artifact (physiological noise). To assist in classification, we also examined the average power spectra and aggregate spatial maps for each component. This was based on the expectations that networks will display peak activations in gray matter, the RSN time course will be dominated by low-frequency fluctuations, and there will be minimal overlap with vascular and ventricular regions on spatial maps [24]. To allow network identification, aggregate spatial maps were correlated with the RSN templates provided by the GIFT toolbox and then visually compared against component spatial maps from [24, 25]. We determined 14 components as either a network or mixture of network and artifact, and 10 components as artifacts.

### Second-level and correlation analyses

A full factorial analysis was completed in SPM5 for each of the components. We generated contrasts between patients and controls groups, co-varying for gender, age, and scan site. From these contrasts, we extracted significant clusters exhibiting peak activity (*t* values) passing FWE-correction, *p <* 0.05. Peak activations at the cluster level were then Bonferroni-corrected with a threshold of *p ≤* 0.002 to correct for multiple comparisons. To examine whether differential functional connectivity between groups was correlated with neuropsychological and psychiatric characteristics, we extracted mean activation values using the Marsbar toolbox in SPM5 (available at http://marsbar.sourceforge.net/). Mean activation values were extracted within a 3-mm spherical ROI from each participant at MNI coordinates corresponding to peak *t* values at voxels from clusters that had passed Bonferroni correction. To account for scanner effects between sites, we conducted a full factorial analysis with the same parameters mentioned above between the patient groups from each site. Of the nine classified networks, we observed very few significant differences between sites, yet nonetheless covaried by scanner site for the aforementioned analysis between 22q11DS and controls described above. Details of these analyses can be found in Additional file 3: Supplementary Material, *Resting-state comparison between scanner sites.*

Distributions of scores for neurocognitive and BPRS measures for both study groups were examined, and Shapiro-Wilks normality tests were conducted. For the neurocognitive measures, nonparametric Spearman correlations were used if the Shapiro-Wilks test indicated that data distributions deviated from normality; otherwise, linear regression analyses were conducted. Since the BPRS is a count variable, a Poisson regression analysis was conducted for each of the four factors of the BPRS.

## Results

**Fig. 1:**
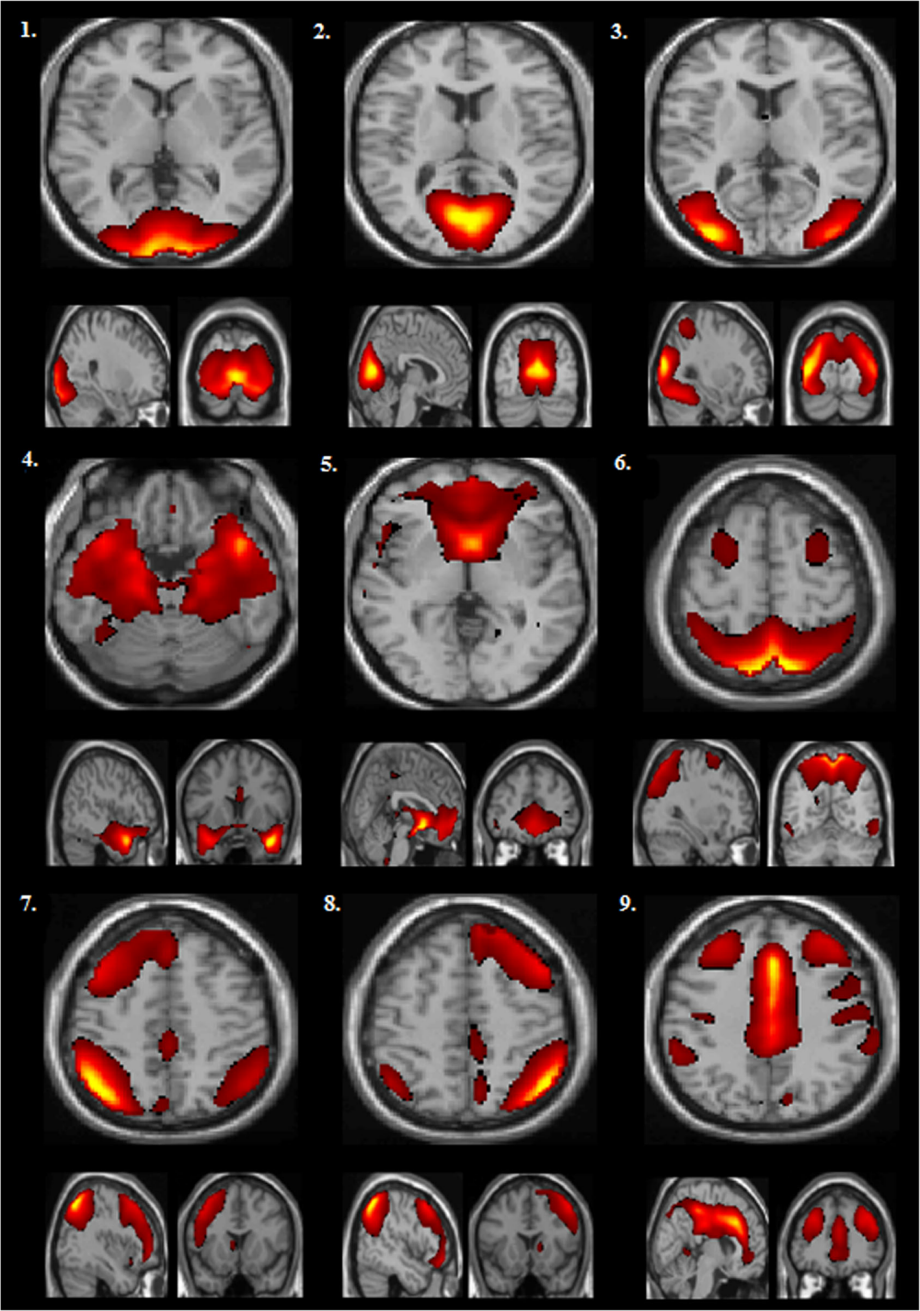
Group ICA was performed on a combined sample of patients and controls. This figure consists of a spatial map depicting (for the full sample) the nine components that subsequently displayed significant group differences in functional connectivity in our full factorial analysis (Bonferroni correction (*p* ≤ 0.002): (*1*) a high-level visual processing network (HLVPN); (*2*) a low-level visual processing network (LLVPN); (*3*) a visual/precuneus network; (*4*) a limbic network (LN); (*5*) a default-mode network (DMN); (*6*) a visual spatial processing network (VSPN); (*7*) a left frontal-parietal network (LFPN); (*8*) a right frontal-parietal network (RFPN); and (*9*) a self-referential network (SN)

After running group ICA, we retained nine networks with significant group differences (Bonferroni-corrected at the cluster level (*p ≤* 0.002). We labeled networks based on terms from the GIFT toolbox and Laird et al. [25]. Group contrasts revealed significant differences in functional connectivity between the combined 22q11DS and control groups (Fig. 1). Decreased within-network connectivity in the 22q11DS group relative to controls (Controls > 22q11DS) was observed in the following networks: high-level visual processing network (HLVPN), subclusters of the low-level visual processing network (LLVPN), visual/precuneus network, left frontal-parietal network (LFPN), right frontal-parietal network (RFPN), and self-referential network (SRN). In contrast, individuals with 22q11DS exhibited greater functional connectivity (22q11DS > controls) in subclusters of the low-level processing network (LLVPN), precuneus/visual network, limbic network (LN), default mode network (DMN), and visuospatial processing network (VSPN). Table 3 displays corrected clusters with their associated MNI coordinates and peak activation (*t* values).

**Table 3:** Comparison of functional connectivity between groups in resting-state networks

Anatomic label	Hem	BA	MNI (x, y, z)	Peak t value^a^	*p* (Bonferroni corr) cluster^a^
High level visual processing network					
Controls vs. 22q11DS					
Lingual gyrus	R	18	21, −87, −9	5.19	<0.001
	L	17	−12, −90, 3	4.67	<0.001
Middle occipital gyrus	R	18	30, −90, −6	4.63	<0.001
	L	18	−21, −93, −3	4.41	<0.001
Inferior occipital gyrus	L	18	−24, −96, −3	4.38	<0.001
Cuneus	R	18	21, −96, 21	3.89	<0.001
	L	17	−9, −99, 15	4.25	<0.001
Low-level visual processing network					
22q11DS vs. controls					
Posterior cingulate	R	29	6, −51, 15	6.07	<0.001
	L	29	0, −42, 15	4.57	<0.001
Controls vs. 22q11DS					
Cuneus	R	18	3, −81, 27	5.86	<0.001
	L	17	−3, −81, 15	4.94	<0.001
Fusiform gyrus	R	37	48, −63, −18	5.36	<0.002
Sub-gyral	R	37	48, −45, −12	4.43	0.002
Visual/precuneus network					
22q11DS vs. controls					
Cuneus	R	17	21, −93, 12	6.12	<0.001
Controls vs. 22q11DS					
Precuneus	L	7	−24, −75, 45	5.12	0.002
Limbic network					
22q11DS vs. controls					
Insula	R	13	36, 33, 6	5.13	<0.001
Inferior frontal gyrus	R	9	36, 15, 18	4.59	<0.001
Default mode network					
22q11DS vs. controls					
Superior parietal lobule	R	7	30, −57, 48	5.07	0.001
Visuospatial processing network					
22q11DS vs. controls					
Precuneus	R	19	15, −81, 45	4.60	<0.001
Cuneus	R	7	27, −81, 39	4.22	<0.001
Left frontal-parietal network					
Controls vs. 22q11DS					
Inferior parietal lobule	L	40	−42, −54, 51	5.72	<0.001
Superior temporal gyrus	L	39	−45, −57, 33	5.14	<0.001
Angular gyrus	L	39	−42 −63 42	4.96	<0.001
Supramarginal gyrus	L	40	−42, −48, 39	4.50	<0.001
Precuneus	L	19	−39, −69, 51	4.24	<0.001
Right frontal-parietal network					
Controls vs. 22q11DS					
Angular gyrus	R	39	48, −57, 45	4.72	<0.001
Superior temporal gyrus	R	39	51, −57, 33	4.29	<0.001
Precuneus	R	39	45, −66, 39	3.87	<0.001
Medial frontal gyrus	R	8	6, 36, 39	4.31	<0.001
Superior frontal gyrus	R	6	30, 12, 66	3.92	<0.001
Sub-gyral	R	6	27, 12, 57	3.23	<0.001
Self-referential network					
Controls vs. 22q11DS					
Insula	R	13	36, 21, 12	5.73	0.002

^a^Peak activation of clusters within resting-state networks between patient and control groups. Clusters depicted in this table passed Bonferroni correction at *p ≤ *0.002; ROIs were extracted for subsequent correlation analyses

From these group contrasts, mean activation values were extracted from 34 ROIs, at MNI coordinates of peak cluster activation, within the clusters that displayed significant differential connectivity between study groups. As described above, we conducted regression analyses to test associations between each of the ROIs and neuropsychological/psychiatric variables.

**Fig. 2:**
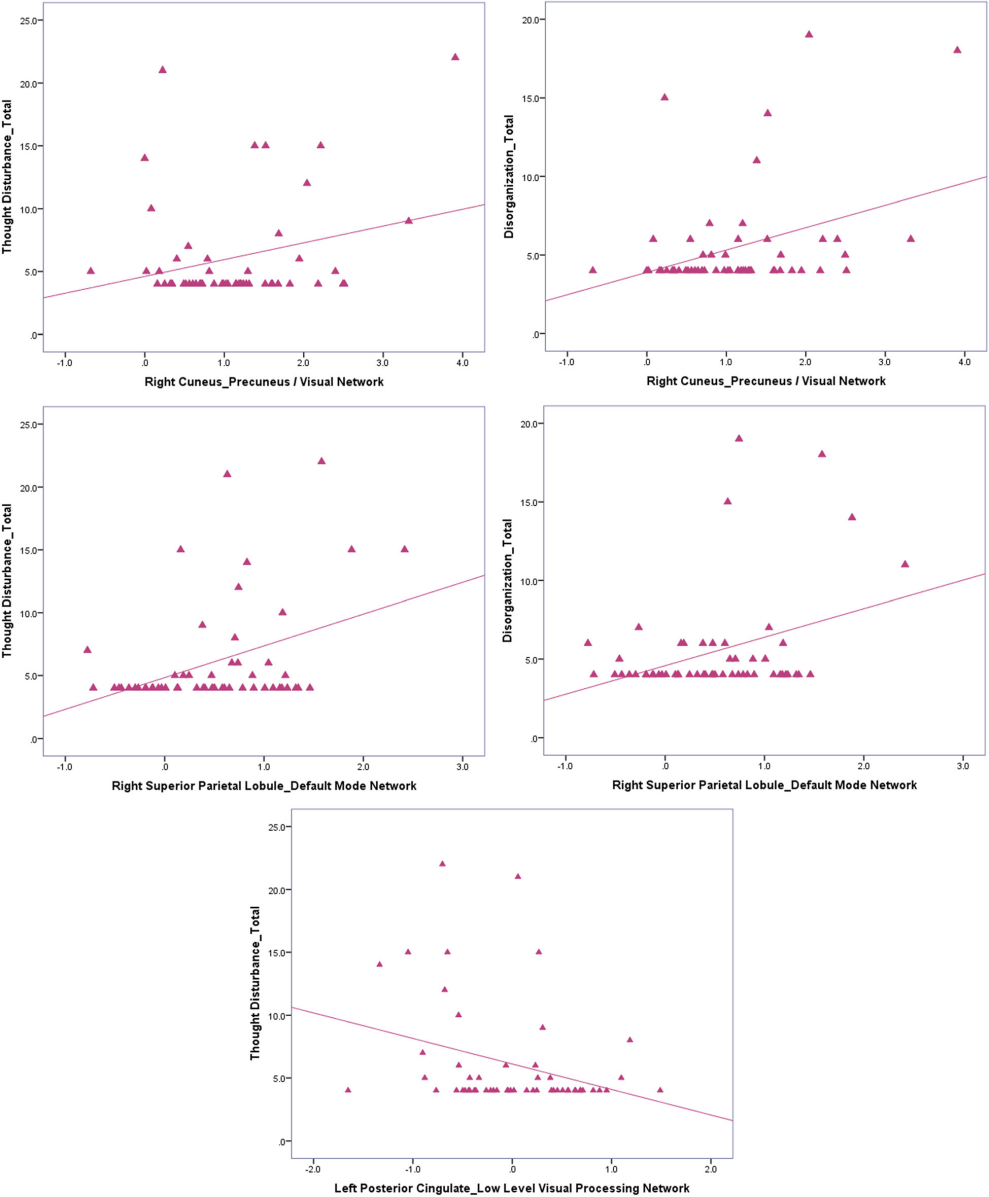
This figure depicts plots representing the association between scores on the thought disturbance and disorganization factors of the BPRS and mean activation values for voxels representing peak activation within the clusters that displayed significant differential connectivity between study groups

Of the four BPRS factors, thought disturbance and disorganization were significantly associated with both increased and decreased functional connectivity within the 22q11DS group after Bonferroni correction, *p ≤* 0.0014 (Fig. 2). Higher total scores of thought disturbance were associated with increased FC within the voxels corresponding to the peak *t* values of subclusters of the visual/precuneus network (right cuneus; *z* = 3.39, *p =* 0.001) and DMN (right superior parietal lobule; *z* = 5.04, *p <* 0.001). In contrast, increased levels of thought disturbance were associated with decreased FC within the left posterior cingulate of the LLVPN (*z* = −3.95; *p <* 0.001).

Higher total disorganization scores were associated with increased FC in the right superior parietal lobule in the DMN (*z* = 3.82, *p <* 0.001) and the right cuneus in the visual/precuneus network (*z* = 3.81, *p <* 0.001).

No correlations between our neuropsychological measures (CVLT list A raw scores and BRIEF GEC scores) and ROI mean signal values passed correction for multiple comparisons, Bonferroni-corrected *p ≤* 0.0014.

### Medication effects

To account for any possible confounding effects from psychotropic medication on the aforementioned results, we conducted another full factorial analysis with the same model parameters described above with the addition of medication as a covariate. A total of 24 patients were currently taking an antipsychotic, mood stabilizer, antidepressant, antianxiety, or psychostimulant medications, and 2 controls were currently taking either an antidepressant/antianxiety or psychostimulant. After correcting for multiple comparisons, group differences in functional connectivity within RSNs remained significant. Individuals with 22q11DS continued to display the same patterns of increased and/or decreased functional connectivity within the HLVPN, LLVPN, visual/precuneus network, limbic network, DMN, VSPN, and LFPN compared to controls. However, we observed that decreased FC in the RFPN and SN in the group with 22q11DS no longer met threshold for statistical significance (Bonferroni correction, *p ≤* 0.002). Decreased FC in the left precuneus of the visual/precuneus network, the left supramarginal gyrus, and precuneus of the LFPN was also no longer significant. Interestingly, after applying the medication covariate, we observed additional results that were not present in the original analysis. In an attention network, the left postcentral gyrus and left superior parietal lobule displayed decreased FC in 22q11DS.

### Group size effects

In order to ensure that the disproportionate sizes of our two study groups (22q11DS: 55; controls: 29) did not affect FC group differences, we split the group of participants with 22q11DS in half (subgroup 1: 27; subgroup 2: 28) and compared each subgroup to the total control sample with a full factorial analysis. *t* tests were conducted to ensure subgroups did not differ in age, gender, full scale IQ, scan site, and comorbid diagnoses. We found that relative to controls, each 22q11DS subgroup displayed similar patterns of decreased/increased FC. These findings also paralleled those of the combined 22q11DS group reported above. Additional details and results of this analysis can be found in Supplementary Material, *Group Size Effects*.

### Comorbidity effects in controls

In order to account for the control participants diagnosed with ADHD, anxiety, and major depressive disorder, we ran a full factorial analysis with all 55 22q11DS participants but excluded the 6 control participants who had a psychiatric diagnosis. Results after Bonferroni correction, *p ≤* 0.002, remained significant, and 22q11DS continued to display the same patterns of decreased/increased FC within the HLVPN, LLVPN, visual/precuneus network, limbic network, DMN, VSPN, LFPN, and RFPN. In the HLVPN, the right cuneus no longer met threshold for significance, as was the case in the LFPN, where the left supramarginal gyrus and left precuneus were no longer significant. We also observed that decreased connectivity in the self-referential network (i.e., right insula) no longer displayed significant differences between study groups. However, decreased FC in the sensorimotor network (i.e., right precuneus) now met threshold for significance, as well as the left cuneus and left lingual gyrus in the visual/precuneus network.

## Discussion

22q11DS is a neurodevelopmental syndrome characterized by deficits in executive functioning, emotion processing, visuospatial reasoning, and working memory. Individuals with this deletion are at risk for a wide range of psychiatric disorders and have an abnormally high susceptibility to developing psychosis. Recent studies indicate that the mean age of onset for a psychotic disorder/schizophrenia in 22q11DS is 17.7/18.4 years [51], which is roughly comparable to idiopathic schizophrenia. Moreover, a large, multi-site study [4] in which data on 1402 participants with 22q11DS from 15 sites were pooled reported that schizophrenia spectrum disorders were present in over 10 % of youth between 13 and 17 years, and in more than 23 % of youth between 18 and 25 years of age. Studies of brain development in typical individuals indicate protracted development of white matter structure (i.e., volumes) and microstructure (i.e., white matter tracts) well into the third and fourth decades of life [52, 53], underlying alterations in and maturation of structural and functional connections throughout the brain. Accordingly, the identification of the nuances in functional connectivity that are associated with prodromal symptoms during this stage of brain maturation may inform our understanding of the contribution RSNs have on the conversion to full-blown psychosis, serving as a potential biomarker for individuals who are at risk.

Using group spatial ICA, we identified RSNs in youth and young adults with 22q11DS between the ages of 17 and 26 years. Of the 33 components tested for group differences, 9 network components contained clusters with significant group differences after correction for multiple comparisons. Group differences between the 22q11DS and controls revealed differential functional connectivity patterns within the following networks: high-level visual processing network, low-level visual processing network, visual/precuneus network, limbic network, default mode network, visuospatial processing network, left and right frontal-parietal networks, and self-referential network. We further observed that mean signal from significant clusters within these networks was associated with items categorized under thought disturbance and disorganization as measured by the BPRS.

Comparing functional connectivity patterns in RSNs between 22q11DS and controls revealed that individuals with 22q11DS exhibited increased connectivity of the right superior parietal lobule in the DMN, which was strongly associated with the presence of symptoms related to thought disturbance and disorganization. The DMN has been implicated in functions involving autobiographic memory, imagination, planning for the future, social cognition, self-referential processing, and emotion processing [1, 54]. The DMN includes interacting subsystems composed of the medial temporal lobe, medial prefrontal cortex, posterior cingulate, precuneus, and lateral parietal cortex [1, 2, 55]. Consistent with reports from Shim and colleagues, increased FC of the DMN was observed in the right superior parietal lobe in participants at ultra-high risk for psychosis [56]. In 22q11DS, both increased and decreased FC of the DMN has displayed correlations with prodromal symptoms in adolescents [31]. Investigation of the DMN in schizophrenia has also demonstrated both increased and decreased FC of the DMN; however, findings of reduced FC within the DMN in schizophrenia have been most commonly reported [2, 57]. Taken together, these results suggest that increased FC in the DMN may be a characteristic of the early/prodromal stages of the disease, and as the disease progresses, reduced functional connectivity between regions of the DMN is a resultant effect. Thus, longitudinal studies of RSNs may be a direction of future investigation.

We found that both increased and, to a lesser extent, decreased FC in visual processing networks were significantly correlated with thought disturbance and disorganization. The LLVPN is typically implicated in orienting toward salient stimuli and has a propensity for simple visual stimuli such as flashing checkerboards [25, 58]. Visual processing deficits have been reported in schizophrenia and are thought to contribute to an altered ability to perceive motion, interpret spatial localization, and detect low-contrast stimuli [59]. The LLVPN is also thought to be a part of the dorsal “where” visual stream [59, 60]. Atypical activity in the dorsal stream is consistent with deficits in visuospatial processing in individuals with 22q11DS [61]. Consistent with findings from Debanné [31], we also observed decreased FC in the HLVPN. The HLVPN is implicated in complex visual stimuli, orthography, and object recognition [25, 62]. This network underlies the ventral “what” visual stream [62]. Dysregulation of the LLVPN (dorsal stream) may influence dysregulation of the HLVPN (ventral stream), as was suggested in an event-related potential study in schizophrenia [63].

In addition, the visual/precuneus network, thought to be associated with mental imagery [64] displayed aberrant FC and was associated with both thought disturbance and disorganization. Specifically, the precuneus is implicated in visuospatial imagery, motor imagery, episodic memory, autobiographical memory, self-processing, and theory of mind [64]. Aberrant connectivity involving the precuneus may account for social dysfunction and visuospatial processing. Taken together with aberrant FC of the visual/precuneus network, dysfunction of these visual streams may contribute to deficits in facial processing, perceptual motor skills and visual-spatial processing, and metacognition, all of which characterize cognitive deficits within 22q11DS and idiopathic schizophrenia [65–67].

Patterns of increased and decreased FC in RSNs that we observed in individuals with 22q11DS are in accordance with previous reports of differential connectivity in 22q11DS. Decreased FC of the HLVPN and increased FC of the VSPN and DMN are consistent with reports from an ICA analysis of RSNs in adolescents (mean age 15.76) with 22q11DS [31]. Consistent with the findings from the same group using a support vector machine classifier approach, the right superior parietal was strongly discriminative of individuals with psychotic symptoms [32]. A previous investigation using a subset of participants included in our UCLA subsample demonstrated that the posterior cingulate, a core hub in the DMN, was found to have decreased FC with other nodes of the DMN and was associated with lower social competence [34]. Although we observed increased FC of the left posterior cingulate to be significantly associated with higher scores of thought disturbance, our results suggest that atypical functional connections of the posterior cingulate may play a role in the emergence of prodromal symptoms, as deficits in social cognition and thought disturbance are two key characteristics of idiopathic schizophrenia [68, 69].

Combined with the findings of reduced gray and white matter in individuals with schizophrenia and 22q11DS, our results suggest that increased FC within RSNs in 22q11DS may serve as a compensatory measure due to aberrant microstructure, as measured by diffusion tensor imaging studies (DTI) [70, 71]. Overcompensation and ultimately dysregulation of these structures due to atypical anatomical connectivity may be responsible for the quintessential cognitive and perceptual deficits observed in populations with psychosis and may serve as an underlying factor in the development of prodromal symptoms. Future investigation of combined structural and functional connectivity data may help to elucidate the underlying nature of increased/decreased FC. Identification of specific genes contributing to these alterations should also be explored.

Atypical FC and activity within RSNs may be attributed to disturbances at the neuronal and neurotransmitter level. Diminished gene dosage within the deleted 1.5–3 megabase (mb) region in 22q11DS has been shown to affect neurogenesis, cortex differentiation, and neuronal migration of interneurons [72]. Moreover, since at least two genes at the 11.2 locus of chromosome 22 are involved in neurotransmitter regulation (e.g., COMT and PRODH), abnormalities at the cellular and neurochemical levels in our sample may also be due to haploinsufficiency of genes from within the1.5–3 mb region in 22q11DS. A study investigating GABA and glutamate in schizophrenia found the balance between excitation and inhibition (glutamate/GABA ratio) and glutamate alone positively correlated with DMN intrinsic functional connectivity, whereas GABA was negatively correlated with functional connectivity in the DMN [73]. It has also been reported that serotonin also has modulatory effects on activity in the DMN [74]. Although these studies only refer to activity within the DMN, our results suggest that further investigation of the underlying neurochemical modulators of RSNs should be explored in 22q11DS.

## Conclusions

The interpretation of these results must also take into consideration the limitations of this study. Scanning protocols, including voxel size and field of view, differed slightly between sites. Therefore, although we covaried by site in our analyses, and follow-up analyses concluded that our results were not driven by scanner differences, we cannot rule out the extent to which those differences may have contributed to some of our results. Within our 22q11DS group, more than half of our sample had a comorbid diagnosis of an anxiety disorder, depression, or ADHD. Symptoms specific to these disorders may have contributed to overall patterns of FC and associations with factors of the BPRS. Since approximately half of our 22q11DS sample was on medication at the time of their scan, we conducted a full factorial covarying for medication. Our results remained significant, though it has been shown that antipsychotics/mood stabilizers affect white matter microstructure [75], cortical thickness, and brain function, where one study found that antipsychotic medication improved performance on a cognitive control task and increased activity in the dorsolateral prefrontal cortex [76]. Antidepressant medication has also been shown to modulate FC in RSNs in individuals with major depressive disorder [77]. Finally, psychostimulant treatment has been found to attenuate deceased FC and affect resting-state perfusion in individuals with ADHD [78]. Therefore, RSN FC patterns that we observed in our sample may have been attenuated due to the use of medication treatment in our 22q11DS group.

To conclude, our study revealed differential functional connectivity patterns within the RSNs of individuals with 22q11DS. Compared to controls, individuals with 22q11DS displayed either decreased or increased within-network connectivity across several RSNs. We observed that increased connectivity in the 22q11DS group significantly correlated with thought disturbance and disorganization factors measured by the BPRS. Taken together, our findings indicate that atypical connectivity within RSNs may serve as a marker of pathology related to prodromal symptoms of psychosis. Elucidation of the relationship between genes and RSNs, as well as longitudinal studies of RSNs, may be a direction of future investigations.

## Additional files

Additional file 1: Table S1. Demographics between sites. Participant characteristics and medical data for the SUNY subsample and UCLA subsample. (DOC 43 kb)

Additional file 2: Table S2. Distributions across sites. Results from the between-site Shapiro-Wilks normality tests for the BPRS, BRIEF-A, and List A raw scores from the CVLT. (DOC 30 kb)

Additional file 3: **Supplementary material.** Details of the analyses comparing RSN functional connectivity between the SUNY and UCLA subsamples and group size effects. (DOCX 21 kb)
