# Supplementary material for: Atypical functional connectivity in resting-state networks of individuals with 22q11.2 deletion syndrome: associations with neurocognitive and psychiatric functioning
Source: J Neurodev Disord. 2016 Jan 21;8:2. doi: 10.1186/s11689-016-9135-z (PMC4743418; doi:10.1186/s11689-016-9135-z)
Supplement: Supplementary file 1 — Demographics between sites. Participant characteristics and medical data for the SUNY subsample and UCLA subsample. (DOC 43 kb) [file 11689_2016_9135_MOESM1_ESM.doc]

Additional file 1: Table S1^a^

Demographics between sites

|  | **SUNY** | **UCLA** |  |
| --- | --- | --- | --- |
|  | 22q11DS Controls | 22q11DS Controls |  |
| Age^b^ | n=39 n=25  20.47(2.05) 20.70(1.22) | n=16 n=4  20.31(2.94) 19.0 (1.83) |  |
| Gender (% male) | 48.7% 60.0% | 50.0% 50.0% |  |
| Full Scale IQ^b^ | 72.77(9.56) 108.76 (9.72) | 74.75(12.78) 113.75(9.91) |  |
| *Psychiatric Diagnosis, n (%)* |  |  |  |
| Psychotic Disorder | 3(7.69%) 0(0%) | 2(12.5%) 0(0%) |  |
| ADHD | 6(15.38%) 3(12.0%) | 3(18.75%) 0(0%) |  |
| Anxiety | 9(23.08%) 3(12.0%) | 6(37.5%) 0(0%) |  |
| Mood Disorders | 6(15.38%) 0(0%) | 3(18.75%) 1(25.0%) |  |
| *Current Medication, n(%)* |  |  |  |
| Antipsychotic/Mood Stabilizer | 5(12.82%) 0(0%) | 2(12.5%) 0(0%) |  |
| Antidepressant/Anti-anxiety | 13(33.33%) 1(4.0%) | 5(31.25%) 0(0%) |  |
| Stimulants | 4(10.26%) 2(8.0%) | 2(12.5%) 0(0%) |  |

^a^ Participant characteristics and medical data between sites. ^b^ Mean and standard deviation are provided for age and full scale IQ.
