# Supplementary material for: Atypical functional connectivity in resting-state networks of individuals with 22q11.2 deletion syndrome: associations with neurocognitive and psychiatric functioning
Source: J Neurodev Disord. 2016 Jan 21;8:2. doi: 10.1186/s11689-016-9135-z (PMC4743418; doi:10.1186/s11689-016-9135-z)
Supplement: Supplementary file 2 — Distributions across sites. Results from the between-site Shapiro-Wilks normality tests for the BPRS, BRIEF-A, and List A raw scores from the CVLT. (DOC 30 kb) [file 11689_2016_9135_MOESM2_ESM.doc]

Additional file 2: Table S2^a^

Distributions Across Sites

| **Tests of Normality** |  |  |  |
| --- | --- | --- | --- |
|  | 22q11DS | Controls |  |
| BPRS total score | <0.001 | N/A |  |
| BRIEF GEF  CVLT List A | 0.101  0.469 | 0.084  0.374 |  |

^a^ Shapiro-Wilks normality tests were conducted on the combined sample, p-values are reported in this table.
