# Supplementary material for: Atypical functional connectivity in resting-state networks of individuals with 22q11.2 deletion syndrome: associations with neurocognitive and psychiatric functioning
Source: J Neurodev Disord. 2016 Jan 21;8:2. doi: 10.1186/s11689-016-9135-z (PMC4743418; doi:10.1186/s11689-016-9135-z)
Supplement: Supplementary file 3 — Supplementary material. Details of the analyses comparing RSN functional connectivity between the SUNY and UCLA subsamples and group size effects. (DOCX 21 kb) [file 11689_2016_9135_MOESM3_ESM.docx]

Additional file 3: Supplementary Material

*Resting-state comparison between scanner sites*

To account for scanner effects between sites, we conducted a subsequent full factorial analysis with the same parameters mentioned above between the patient groups from each site. Since we only utilized 4 control participants from the UCLA subsample, there was not enough power to conduct a full factorial between control groups for each site. Of the nine classified networks, we observed significant differences in the bilateral cuneus of the LLVPN, left supramarginal gyrus and precuneus in the LFPN, and right middle frontal gyrus in the RFPN. Out of all of these structures, only the right cuneus displayed significant differences between study groups at the same peak MNI coordinates where differential FC was observed between patients and controls. Differential FC of the supramarginal gyrus and precuneus displayed significant differences between patients and controls as well, however at different peak voxels than those described above. The middle frontal gyrus did not display significant differences between study groups and was not used in any regression analyses.

*Group size effects*

In order to ensure that the disproportionate sizes of our two study groups did not affect FC group differences, we divided the group of participants with 22q11DS in half, and compared each subgroup to the total control sample by conducting a full factorial analysis with the same parameters used for the between group differences for the combined SUNY and UCLA subsamples in our second level analysis. 22q11DS groups did not differ in age, gender, or full scale IQ. Comorbid diagnoses of anxiety, major depressive disorder, ADHD, and psychosis were matched for each group. We also included exactly half of the UCLA subsample (8 participants with 22q11DS) in each group. A full factorial between 22q11DS groups was conducted to determine any differences in FC of RSNs. Significant between group differences (Bonferroni corrected, p≤0.001) were observed in the HLVPN (right middle occipital gyrus, right lingual gyrus, right inferior occipital gyrus, right fusiform gyrus) and LLVPN (bilateral cuneus).

Subsequently, 2 full factorials were then carried out comparing FC of each 22q11DS group to that of the control group. For both analyses, individuals with 22q11DS continued to display the same patterns of increased/decreased FC in the HLVPN, LLVPN, visual/precuneus network, limbic network, VSPN, LFPN, and RFPN. However, by splitting the 22q11DS group, we observed that the first 22q11DS subgroup displayed increased FC in the sensorimotor network and decreased FC in the attention network, that now reached significance (Bonferroni corrected, *p≤* 0.002), whereas the LFPN no longer met threshold for statistical significance. Splitting the 2 groups also led to the loss of the self-referential network and DMN, which no longer met threshold for significance.
